# Ultrasonography of Diaphragm to Predict Extubation Outcome

**DOI:** 10.7759/cureus.36514

**Published:** 2023-03-22

**Authors:** Ishita Raj, Suresh Kumar Nagaiah

**Affiliations:** 1 Anaesthesiology, Sri Devaraj Urs Medical College, Kolar, IND; 2 Anesthesia and Critical Care, Sri Devaraj Urs Medical College, Kolar, IND

**Keywords:** weaning from mechanical ventilator, intensive care unit, ventilator weaning, mechanical ventilation, ultrasonography of diaphragm, spontaneous breathing trial, extubation

## Abstract

Background and objectives

An intensivist’s decision, when it comes to weaning off a patient from mechanical ventilation and extubation, is based on many criteria. Problems can be associated with both prolonged ventilation and early extubation. Therefore, for optimal functioning of the diaphragm, the primary inspiratory muscle, it is important to resume spontaneous ventilation after extubation irrespective of the cause of respiratory failure. Thus, diaphragmatic parameters can prove useful in predicting the rate of success of extubation. However, till date in our institute, extubation has been done using only the standard parameters; hence, in this study, diaphragmatic parameters obtained by ultrasonography-diaphragmatic thickening fraction (dTF) and diaphragmatic excursion (DE) have been studied to predict extubation outcome.

The objectives of this study are to (1) measure the diaphragmatic thickening fraction (dTF) in % and diaphragmatic excursion (DE) in cm before and after the spontaneous breathing trial (SBT) and (2) compare dTF and DE with standard extubation parameters in order to predict extubation outcome.

Materials and methods

This is a prospective, double-blind, observational study. The number of patients involved was 41. After obtaining ethical committee clearance, informed consent was taken from the patients’ attendants. In this study, we divided doctors into the treating team and the research team. The treating team comprised the primary doctors working in the intensive care unit (ICU), and they evaluated the patients’ readiness for pressure support ventilation. The research team performed diaphragmatic ultrasonography on those patients who met the inclusion criteria. The treating team was blinded to the dTF and DE results obtained by the research team. Prior to extubation, all the patients had to satisfy the standard extubation criteria followed at R. L. Jalappa Hospital and Research Centre (RLJH), Kolar

Results

We observed that 68.29 (%) of the patients with normal dTF and DE values and 21.95(%) with slightly lower dTF and DE values were extubated successfully, and 7.31 (%) with normal dTF and DE values were reintubated; 2.43 (%) were extubated onto non-invasive ventilation (NIV).

Conclusion

From our study, we have concluded that bedside ultrasonography of the diaphragm, that is the measurement of diaphragm for dTF and DE always compliments the standard criteria for extubation and can be used for weaning the patients from mechanical ventilation, as bedside ultrasonography is not only easy and convenient but also a reliable parameter in predicting the outcome of weaning, however, it cannot be used as a sole criteria.

## Introduction

Mechanical ventilation is indicated for the treatment of acute respiratory failure triggered by a number of underlying medical conditions or trauma or in the postoperative period in the intensive care unit (ICU). It is the main responsibility of an intensivist to render adequate mechanical ventilation to the patient and to decide when to wean the patient off the ventilator; this is a challenging task [1].

When assessing if a patient can be effectively extubated, timing is crucial. Weaning off mechanical ventilation and extubation are always challenging for an intensivist because both extended ventilation and premature extubation are linked to a variety of comorbidities, resulting in increased patient healthcare costs and a longer stay in the ICU. Prolonged ventilation is associated with complications such as ventilator-associated pneumonia, barotrauma, and atrophy of the respiratory muscles, while premature extubation can result in hypoxia, hypercarbia, and increased respiratory and cardiac distress [2]. Hence, weaning should be considered once the patient is able to sustain spontaneous breathing. This decision is made based on the correction of the underlying pathology, improvement in the patient’s Glasgow Coma Scale (GCS) score, airway protective mechanism, ability to cough, hemodynamic stability, and absence of other acute problems [3].

Till date, no study utilizing diaphragmatic ultrasonography has been conducted at R. L. Jalappa Hospital and Research Centre (RLJH) to predict extubation outcomes. Diaphragmatic parameters can prove useful to predict the rate of success of extubation, hence, we conducted this study to evaluate the following diaphragmatic parameters, along with the standard extubation parameters, to predict extubation outcome. We took the measurements of the diaphragmatic thickening fraction (dTF) in % and diaphragmatic excursion (DE) in cm before and after the spontaneous breathing trial (SBT).

Routinely, a checklist is followed to identify candidates who are fit for spontaneous breathing trial (SBT). The respiratory criteria for being considered fit are as follows: partial pressure of oxygen (PaO2)/fraction of inspired oxygen (FiO2) > 150-200 mmHg, FiO2 ≤ 50%, and positive end expiratory pressure (PEEP) ≤ 8 cm H2O. With partial pressure of carbon dioxide (PaCO2) at normal or baseline levels, the patient is able to initiate an inspiratory effort. Cardiovascular criteria for inclusion: no evidence of myocardial ischemia, heart rate ≤ 70-90 beats/min, and adequate blood pressure with minimal or no vasopressors [4]. Moreover, appropriate mental status is required, which is ensured by checking if the patient is arousable or whether the GCS score is ≥ 13. Finally, there should be no correctable comorbidities, no fever, and no significant electrolyte abnormalities [5,6].

Successful weaning is defined as the absence of any form of ventilatory support for 48 h following the extubation. During this period, while spontaneous breaths remain unassisted by mechanical ventilation, supplemental oxygen, bronchodilators, pressure support ventilation (PSV), or continuous positive airway pressure (CPAP) can be used to support and maintain adequate spontaneous ventilation and oxygenation. Optimal functions of the diaphragm, which is the primary muscle of inspiration, are important to resume spontaneous ventilation after extubation irrespective of the cause of respiratory failure.

SBT is defined as one of the most significant tests to determine if patients can be successfully extubated and weaned from mechanical ventilation [4]. Low-level pressure support (PS) and CPAP may be used along with SBT. When the decision is made to wean, the patient is removed from full ventilatory support and placed on spontaneous breathing mode for up to 30 min via the ventilator or T-Piece. Oxygen and low PS (up to 8 cm H2O for adults and 10 cm H2O for pediatrics) are used to supplement oxygenation and augment spontaneous breathing. Criteria for conducting SBT are a normal respiratory pattern (absence of rapid shallow breathing), adequate gas exchange, and hemodynamic stability. When the patient passes SBT and when level of blood gasses and vital signs are satisfactory, extubation is considered.

SBT failure criteria are as follows: PaO2 < 60 mmHg on FiO2 > 50%; oxygen saturation (SaO2) < 90% on FiO2 > 50%; PaO2 > 50 mmHg or an increase in PaCO2 > 8 mmHg from baseline of SBT; potential of hydrogen (pH) < 7.32; Rapid Shallow Breathing Index (RSBI), which is defined as the ratio of respiratory frequency to tidal volume (f/TV), > 100 breaths/min/l; respiratory rate measured as frequency (f) > 35 breaths/min or increase by > 50% from baseline of SBT; heart rate >140 beats/min or increase by > 20% from baseline of SBT; systolic blood pressure (BP) >180 mmHg or increase by > 20% from baseline of SBT; systolic BP < 90 mmHg or presence of cardiac arrhythmias.

## Materials and methods

This is a prospective, double-blind, observational study. After obtaining ethical approval from the institutional ethics committee, Sri Devaraj Urs Medical College, Kolar (approval number: SDUMC/KLR/IEC/127/2019-20), the study was conducted on 41 patients who were admitted to the ICU with requirement for mechanical ventilation and who satisfied the inclusion criteria at R. L. Jalappa Hospital and Research Centre (RLJH), Tamaka, Kolar, during the period from January 2020 to May 2021. Inclusion criteria included patients over the age of 18 who were on mechanical ventilation because of respiratory failure and were ready to transition to PSV. The study excluded patients with spinal cord injury, arrhythmias and hemodynamic instability, pneumothorax, pneumomediastinum, thoracotomy, chest tube, chest injuries that prevent ultrasonography, pleural lesions or pleurodesis, pregnancy, or a poor ultrasonography image. Informed consent was taken from the patients’ attendants. A pro forma was used to record the results.

Necessary investigations such as blood hemoglobin (gram/decilitre) and arterial blood gas (ABG) were done prior to subjecting the patient to PSV; ABG was done immediately before extubation. In this study, we divided doctors into the treating team and the research team. The treating team comprised the primary doctors working in the ICU, and they evaluated the patients’ readiness for PSV. The research team performed diaphragmatic ultrasonography on those patients who met the inclusion criteria. The treating team was blinded to the dTF and DE results obtained by the research team.

Prior to extubation, all the patients had to satisfy the standard extubation criteria followed at RLJH. The criteria included 2 h of PSV with PEEP = 5 cm of H2O and PS = 8 cm of H2O. They also included the following measurements: respiratory rate (RR) < 30, exhaled TV > 6 ml/kg ideal body weight, RSBI < 105 breaths/min/l, and leak test > 100 ml difference in TVs.

**Figure 1:**
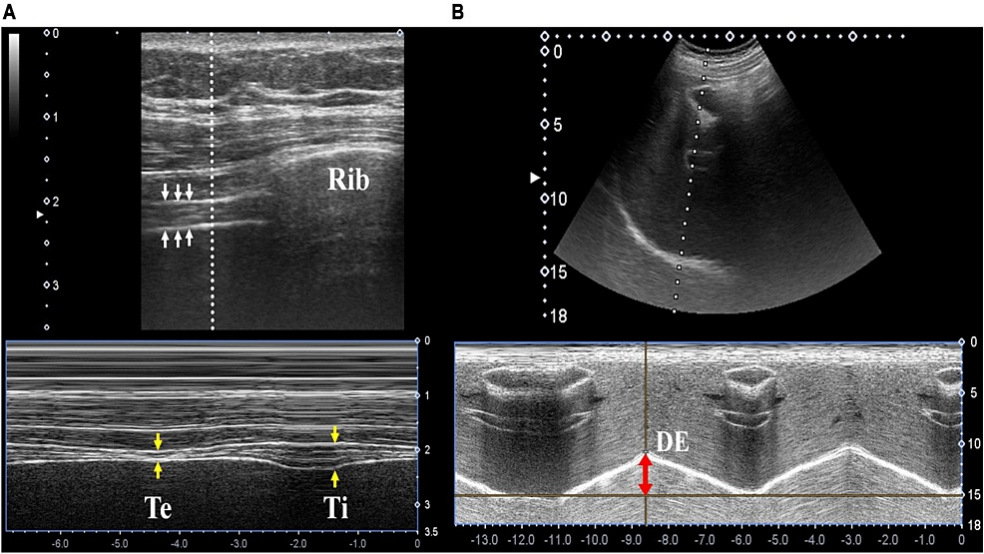
Measurement of the diaphragmatic thickening fraction and diaphragmatic excursion. (A) Diaphragmatic thickening fraction and (B) Diaphragmatic excursion (DE) Te- diaphragm thickness measurement during expiration; Ti- diaphragm thickness measurement during inspiration.

The research team scanned the diaphragm for dTF and DE twice before extubation (Figure 1). The first scan was done immediately prior to subjecting the patient to PSV, and the second scan was done prior to extubation. In the diaphragmatic ultrasonography examination, dTF was measured via the M-mode using a 7-10 MHz linear probe placed on the anterior to a mid-axillary line between the eighth and tenth intercostal spaces. DE was measured in the mid-clavicular line via the M-mode using a 2-5 MHz curvilinear probe during the respiratory cycle. Both these measurements were taken with the patient in a semi-decubitus position (30-degree head-end elevation).

The sonographer captured three measurements each time, and the mean of these three values was taken and incorporated into the formula for dTF = (Diaphragmatic thickness at end-inspiration−Diaphragmatic thickness at end-expiration)/Diaphragmatic thickness at end-expiration*100); a value above 35% was considered suitable for weaning.

DE measured the distance that the diaphragm was able to move during the respiratory cycle. DE = Movement of the diaphragm during inspiration-Movement of the diaphragm during expiration. DE between 2 and 5 cm was considered normal, and a value greater than 1 cm was considered suitable for extubation. The parameters observed were ABG, peripheral capillary oxygen saturation (SpO2), Respiratory rate (RR), RSBI, and exhaled tidal volume (TV).

Sample size

The sample size was calculated based on the correlation between the DE and dTF in a 2018 study [3]; with desired confidence level of 95% and a precision of 10% and assuming the expected proportion of 0.88, the total sample size was calculated as 41 patients.

Statistical analysis

The information gathered was coded and entered into an Excel database. All the quantitative measures were presented in terms of mean + standard deviation (SD) and confidence interval (CI); the qualitative measures were presented by proportions and CI. Sensitivity, specificity, positive predictive value (PPV), negative predictive value (NPV), receiver operating characteristic (ROC), and area under the curve (AUC) analyses were used. The Chi-square test was used to determine the significance of difference in proportions. A P value < 0.05 was considered to be statistically significant.

## Results

Table 1 shows the minimum, maximum, mean, and standard deviation (SD) values of the standard parameters for extubation. It was found that RR was 20±3.83, SpO2 was 98.83±1.27, minute ventilation (MV) was 8.36±1.92, and TV was 479.71±28.85.

**Table 1:** Standard parameters for extubation RR-respiratory rate; cpm-cycles per minute; SpO2-saturation of oxygen; MV-Minute ventilation; l-liter; TV-Tidal volume; ml-millilitre; * Moderately significant ( P value:0.01<P < 0.05) ** Strongly significant (P value : P<0.01)

Standard parameter for extubation	Min	Max	Mean	SD
RR (cpm)	14.00	30.00	20.06	3.83
SpO2 (%)	96.00	100.00	98.83	1.27
MV (l)	4.50	11.90	8.36	1.92
TV (ml)	400.00	500.00	479.71	28.85

Table 2 shows ABG analysis values recorded prior to PS as well as prior to extubation. FiO2 value was considered as strongly significant with a P value<0.001, followed by pH value with a P value of 0.018.

**Table 2:** ABG assessment prior to PS and extubation ABG-arterial blood gas; PaO2-partial pressure of oxygen; PaCO2-partial pressure of carbon dioxide; FiO2-fraction of inspired oxygen, PS-pressure support

ABG						Prior to PS	Prior to extubation	Difference	t value	P value
ABG	Prior to PS	Prior to extubation	Difference	t value	P value	7.40±0.10	7.44±0.07	–0.038	–2.458	0.018*
pH	7.40±0.10	7.44±0.07	–0.038	–2.458	0.018*	120.76±48.55	126.32±58.74	–5.561	–0.663	0.511
PaO2	120.76±48.55	126.32±58.74	–5.561	–0.663	0.511	41.83±8.86	35.49±1.87	6.341	4.575	<0.001**
FiO2	41.83±8.86	35.49±1.87	6.341	4.575	<0.001**	37.95±14.21	34.71±7.25	3.244	1.657	0.105
PaCO2	37.95±14.21	34.71±7.25	3.244	1.657	0.105					

Table 3 shows the RSBIs of the 41 patients studied, out of which 13 were female and 28 were male. RSBI is considered normal if the value is <105. The lower the RSBI value, the better the chances for extubation, and in this study, it was found that the majority of the patients, i.e., 17 patients (41.5%), had RSBI between 20 and 30 bpm/l.

**Table 3:** RSBI No.-number; RSBI-rapid shallow breathing index

RSBI	No. of patients	%
20–30	17	41.5
31–40	15	36.6
41–50	9	21.9
Total	41	100.0

Table 4 shows that three values of DE were taken prior to PS as well as prior to extubation; among these, it was observed that the third value recorded prior to PS,1.61±0.35, was strongly significant with a P value <0.001,followed by the first value recorded prior to PS, 1.68±0.41, which was considered moderately significant with a P value of 0.018. Most of the values obtained for DE prior to PS as well as prior to extubation were found to be above 1.5 cm on the graph. 

**Table 4:** DE assessment prior to PS and extubation DE-diaphragmatic excursion; PS-pressure support

Observation	Prior to PS	Prior to extubation	Difference	t value	P value
1^st^	1.68±0.41	1.81±0.27	–0.127	–2.458	0.018*
2^nd^	1.61±0.31	1.84±0.30	–0.225	–0.663	0.511
3^rd^	1.61±0.35	1.85±0.27	–0.235	4.575	<0.001**
Average	1.63±0.33	1.83±0.26	–0.197	1.657	0.105

Table 5 shows three dTF values recorded prior to PS and prior to extubation and the average values for the three observations made. It was found that the third value of dTF,55.52±21.96, and the average value, 54.96±17.94,both obtained prior to PS, were strongly significant with P value of 0.001.

**Table 5:** dTF assessment prior to PS and extubation dTF-diaphragmatic thickening fraction; PS-pressure support

Observation	Prior to PS	Prior to extubation	Difference	t value	P value
1^st^	57.30±26.02	62.86±24.71	–5.561	–1.408	0.167
2^nd^	57.00±19.86	62.67±18.52	–5.676	–1.850	0.072+
3^rd^	55.52±21.96	66.22±17.14	–10.700	–3.742	0.001**
Average	54.96±17.94	63.87±17.70	–8.908	–3.718	0.001**

## Discussion

Mechanical ventilation is considered a life-saving procedure in acute respiratory failure and trauma. However, both prolonged mechanical ventilation and early extubation are regarded as equally bad, as both can cause increased mortality and morbidity in the ICU. Prolonged ventilation is linked to a number of serious problems, including nosocomial pneumonia, barotrauma, airway difficulties, and respiratory muscle atrophy, and accounts for a considerable portion of the costs incurred by patients in critical care units [7].

Similarly, early discontinuation of mechanical ventilation can also lead to multiple problems. Patients may hyperventilate due to hypoxia, pain, anxiety, or inappropriate ventilator settings. Early signs of weaning failure include the use of accessory muscles, paradoxical abdominal movements, tachypnea, dyspnea, chest pain, asynchrony, and diaphoresis. Weaning from mechanical ventilation is a difficult decision because it entails switching from mechanical breathing support to the patient’s own respiratory drive. This transition is very critical and needs an accurate, intense, confident decision based on trusted, validated clinical, radiological, and laboratory parameters to avoid the risk of weaning failure [8]. Zhou et al. [9] defined weaning failure as the requirement of mechanical ventilation protocols within 48 h after extubation.

In other institutes apart from the standard parameters, nowadays, diaphragmatic ultrasonography is used to predict extubation outcomes because the diaphragm is considered the major respiratory muscle. Ultrasonography is routinely used as a non-invasive bedside tool to assess diaphragmatic function and lung parenchyma status. It can assess the percentage change in diaphragmatic thickness from expiration to inspiration (dTF) and the amplitude of diaphragmatic dome movements during the respiratory cycle (DE), which are indicators of the strength of diaphragmatic contractions [10].

In this study, two values of ABG parameters were documented; one was taken prior to the PS trial, and one was taken just before extubation. Among all the ABG parameters, only four were taken into consideration: pH, PaO2, PaCO2, and FiO2. Standard variables considered for extubation are RR, SpO2 (%), MV, TV, and RSBI. In this study, it was found that the mean value of RR was 20 beats/min, the mean of SpO2 was 98%, the mean of MV was 8.36 l, and the mean of TV was 479 ml. The RSBI in the majority of the patients (41.1%) was found to be between 20 and 30 breaths/min/l. After considering all the routine parameters and ultrasonography measurements, we needed to conclude if extubation was a success.

It was observed from our study that out of 41 patients, out of which 28 were male and 13 were female, on whom the study was done, 37 patients were successfully extubated, had more than the normal values considered for dTF. In this study, we measured only the right side of the diaphragm and came to the conclusion that dTF >35% can be considered for extubation. In the study conducted by Gottesman and McCool [11], the mean dTF of the right hemidiaphragm was 35.4%; these values correlated well with the values obtained in our study.

Among the 41 patients, 37 patients were successfully extubated with DE>1 cm.Hence we concluded that DE>1 cm could be considered for extubation. In the study conducted by Sabri et al. [12], excursions in normal breathing ranged from 0.9 to 3.6 cm, with a mean of 2 cm; the mean excursion for the right hemidiaphragm was 1.85 cm; These values were also similar to the values obtained in our study.

However, nine out of these 37 patients despite having a slightly lower value of dTF (34%) and only one out of the nine had dTF of 28% were also extubated. Despite having a dTF of 34%, all these eight patients had more than 1 cm value for DE. Hence the value of dTF of 34% in all these eight patients could be neglected as it was at the borderline. The only patient with a dTF of 28% could be due to poor ultrasound imaging. We also observed that only three patients were re-intubated. However, out of these three patients that were re-intubated two of them had type II respiratory failure and only one patient was re-intubated due to airway compromise. One patient was extubated onto non-invasive ventilation (NIV), that was also later weaned off and patient was doing well.

Overall, the findings of previous studies were inconsistent and lacked statistical power, and the clinical significance of DE and dTF still remained debatable. For example, Lerolle et al. [13] demonstrated that DE correlated well with transdiaphragmatic pressure and suggested that DE could reflect diaphragmatic dysfunction, but this study did not comment on the use of dTF. Meanwhile Umbrello et al. [14] believed that dTF, rather than DE, was a more reliable indicator of respiratory effort and diaphragmatic contractile function. Hence in our study, we investigated both DE and dTF and checked whether both parameters, in combination with standard parameters, can help predict extubation outcome and concluded that DE and dTF could be used along with the routine parameters for regular extubation, but they cannot be the sole indicator. According to Boussuges et al. [15], DE and dTF investigation allows paralysis to be diagnosed, hence helping in predicting the extubation outcome [15].

This study can only be done in a tertiary care hospital with a dedicated critical care unit for trauma, surgical and medical patients. Our ICU had an infrastructure that ensured comprehensive care and provided bedside ultrasonography for the assessment of each patient who was suitable for our study. However, this study also has its limitations. One of the limitations is that it is difficult to perform ultrasonography in obese and pregnant patients, as blurry images may be obtained. Another limitation is the small sample size of this study.

## Conclusions

Based on our findings, it can be concluded that bedside ultrasonography of the diaphragm always complements the standard criteria for extubation and can be used to predict extubation outcomes because it is a point-of-care assessment that is repeatable, reliable, and free of complications. In our study, we measured the diaphragmatic thickening fraction (dTF) and diaphragmatic excursion (DE) for all those patients who were planned for weaning from mechanical ventilation and who met the inclusion and exclusion criteria. These measurements were taken on a trial basis subjecting the researchers to a blind study and were not included along with the standard protocol followed for extubation.

It was observed from our study that out of 41 patients on whom the study was done, 37 patients were successfully extubated, had more than the normal values considered for dTF and DE. Hence, dTF and DE can be used along with the routine parameters for regular extubation as bedside ultrasonography is not only easy and convenient but also a reliable parameter in predicting the outcome of weaning. However, to come to any conclusion regarding the ultrasonography of the diaphragm that can be considered as a sole criteria to predict successful extubation, we needed a larger group of study with a bigger sample size.
